# A Computational Model for the Automatic Diagnosis of Attention Deficit Hyperactivity Disorder Based on Functional Brain Volume

**DOI:** 10.3389/fncom.2017.00075

**Published:** 2017-09-08

**Authors:** Lirong Tan, Xinyu Guo, Sheng Ren, Jeff N. Epstein, Long J. Lu

**Affiliations:** ^1^Division of Biomedical Informatics, Cincinnati Children's Hospital Research Foundation Cincinnati, OH, United States; ^2^Department of Electrical Engineering and Computing System, University of Cincinnati Cincinnati, OH, United States; ^3^Department of Mathematical Sciences, McMicken College of Arts and Sciences, University of Cincinnati Cincinnati, OH, United States; ^4^Department of Pediatrics, Cincinnati Children's Hospital Research Foundation Cincinnati, OH, United States; ^5^School of Information Management, Wuhan University, Wuhan Hubei, China; ^6^Department of Environmental Health, College of Medicine, University of Cincinnati Cincinnati, OH, United States

**Keywords:** attention deficit hyperactivity disorder, automatic diagnosis, functional volume, machine learning, support vector machine

## Abstract

In this paper, we investigated the problem of computer-aided diagnosis of Attention Deficit Hyperactivity Disorder (ADHD) using machine learning techniques. With the ADHD-200 dataset, we developed a Support Vector Machine (SVM) model to classify ADHD patients from typically developing controls (TDCs), using the regional brain volumes as predictors. Conventionally, the volume of a brain region was considered to be an anatomical feature and quantified using structural magnetic resonance images. One major contribution of the present study was that we had initially proposed to measure the regional brain volumes using fMRI images. Brain volumes measured from fMRI images were denoted as *functional volumes*, which quantified the volumes of brain regions that were actually functioning during fMRI imaging. We compared the predictive power of functional volumes with that of regional brain volumes measured from anatomical images, which were denoted as *anatomical volumes*. The former demonstrated higher discriminative power than the latter for the classification of ADHD patients vs. TDCs. Combined with our two-step feature selection approach which integrated prior knowledge with the recursive feature elimination (RFE) algorithm, our SVM classification model combining functional volumes and demographic characteristics achieved a balanced accuracy of 67.7%, which was 16.1% higher than that of a relevant model published previously in the work of Sato et al. Furthermore, our classifier highlighted 10 brain regions that were most discriminative in distinguishing between ADHD patients and TDCs. These 10 regions were mainly located in occipital lobe, cerebellum posterior lobe, parietal lobe, frontal lobe, and temporal lobe. Our present study using functional images will likely provide new perspectives about the brain regions affected by ADHD.

## Introduction

Attention Deficit Hyperactivity Disorder (ADHD) is a psychiatric disorder characterized by clinical symptoms of inattention, impulsivity, and hyperactivity. This condition affects 5–8% of school age children, and usually persists into adolescence and adulthood. Clinical diagnosis of ADHD is based on behavioral information gathered from parents and school. Depending on the number and type of symptoms, a child can be diagnosed with one of three ADHD presentations: primarily inattentive (ADHD-I), primarily hyperactive (ADHD-H) or combined subtype (ADHD-C; American Psychiatric Association, 2013). Despite its high prevalence, the precise etiology and pathogenesis of ADHD remains unclear.

In recent years, magnetic resonance imaging (MRI) studies of patients with ADHD have demonstrated possible physiological underpinnings of the disorder. Modern machine learning techniques with a large-scale dataset may help to identify reliable neuroimaging biomarkers, which may offer some clues to the physiological basis of the disorder. Toward this aim, the ADHD-200 Consortium organized the ADHD-200 global competition, making hundreds of anatomical and functional images publicly available (Consortium, 2012). The ADHD-200 global competition released the demographic and clinical data, anatomical, and resting-state functional MR images for 973 participants accumulated from eight independent sites: Bradley Hospital/Brown University, Kennedy Krieger Institute, NeuroIMAGE Sample, New York University Child Study Center, Oregon Health & Science University, Peking University, University of Pittsburgh, and Washington University. In order to bring the ADHD-200 global competition to a wider audience, the Neuro Bureau performed the preprocessing systematically on the Athena computer cluster at Virginia Tech's ARC and made the preprocessed data openly downloadable.

Spontaneous low frequency fluctuations in blood oxygen level dependent (BOLD) activity are a fundamental feature of the brain at rest. The relative magnitude of these fluctuations is usually measured by the amplitude of low-frequency fluctuations (ALFF; Zang et al., 2007) or fractional amplitude of low-frequency fluctuations (fALFF; Zou et al., 2008). ALFF is a voxel-by-voxel calculation of the power spectrum of the BOLD fMRI time series. fALFF is the ratio of power spectrum of low-frequency (0.009–0.08 Hz) to that of the entire frequency range. ALFF/fALFF was widely used to study the abnormal spontaneous brain activities in various diseases, such as schizophrenia (Hoptman et al., 2010), amnestic mild cognitive impairment (Han et al., 2011, 2012), Parkinson's disease (Skidmore et al., 2013), and major depressive disorder (Jiao et al., 2011; Wang et al., 2012). Studies in ADHD also reported decreased/increased ALFF/fALFF in various brain regions (Zang et al., 2007; Yang et al., 2011; An et al., 2013). fALFF has been evaluated in the ADHD-200 competition dataset. In particular, Cheng et al. used the fALFF coefficient at each voxel as an indicator of ADHD status and then applied two-sample *t*-test to select significant voxels for subsequent model training (Cheng et al., 2012). Combining fALFF with regional homogeneity (ReHo) and information from brain networks, they achieved a cross-validated accuracy of 76.15% for the classification of ADHD patients vs. healthy controls on the dataset collected from Peking University. Sato et al. (2012b) applied the brain parcellation defined by CC400 atlas, which was generated by the Neuro Bureau via a two-level spatially constrained spectral clustering algorithm (Craddock et al., 2012) and divided the brain into 351 regions. The mean fALFF within each brain region was calculated and used as a predictor in their classification model. Based on the whole ADHD-200 dataset, Sato et al. suggested that the combination of fALFF and ReHo contained information to distinguish ADHD patients from healthy controls, but with limited discriminative power. The CC400 atlas was publicly available at the competition website, and was widely used in studies on the ADHD-200 dataset (Colby et al., 2012; Dai et al., 2012; Sato et al., 2012a,b, 2013).

Through a close examination, we observed that there were many voxels covered by the CC400 atlas but their fALFF coefficients were zero. According to the definition of fALFF (Zou et al., 2008), a voxel exhibited zero fALFF coefficient only when there was no low-frequency fMRI signal (0.009–0.08 Hz) at this voxel. On the other hand, CC400 atlas was supposed to cover only voxels within the brain, which were assumed to have functional activities. Thus, voxels covered by CC400 atlas should not have zero fALFF coefficients, which contradicted our actual observation. To uncover the causes underlying the voxels with zero fALFF coefficients, we investigated into the preprocessing steps of the Athena pipeline. The Neuro Bureau generated an fMRI mask for each individual using the AFNI program “3dAutomask,” which took an fMRI image as input and output a brain-only mask. Specifically, voxels within the brain were marked as “1” while voxels outside the brain were marked as “0” in this brain-only mask. The Neuro Bureau then applied the spatially normalized fMRI mask to the calculation of fALFF maps. Voxels marked as “0” in the fMRI mask were assigned zero fALFF coefficients automatically, because those voxels were outside of the brain. For more details, please refer to the preprocessing codes provided by the Neuro Bureau on their website as well as the AFNI manual.

**Figure 1:**
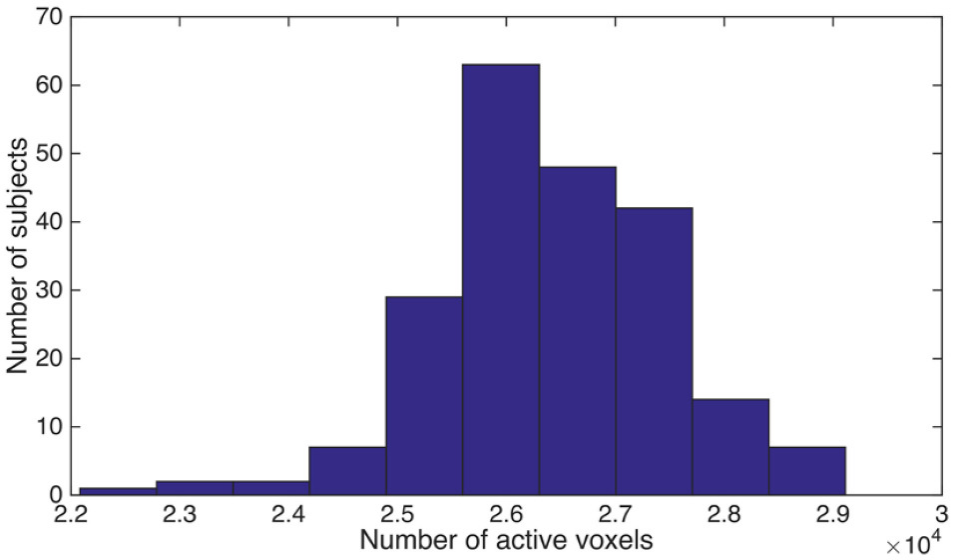
We counted the number of “1” voxels within the fMRI mask for each subject, and summarized the distribution of subjects using a histogram. Horizontal axis is the total number of “1” voxels for a subject. Vertical axis is the number of subjects.

As we described above, the spatially normalized fMRI mask was generated for each subject individually. Since it was a brain-only mask, the count of “1” (or within-brain) voxels could be used as a measure for the volume of brain tissue that was functionally active. We named this brain volume as ***functional volume***, which was the counterpart of ***anatomical volume***. We observed substantial variance for the functional brain volume across different subjects, although the brains from different subjects had been spatially normalized and were expected to be almost perfectly aligned. The individual differences were illustrated in Figure 1.

Conventionally, volume of a brain region was considered to be an anatomical feature and usually calculated based on anatomical magnetic resonance images. To the best of our knowledge, functional volume was initially proposed by us in the current study. We first compared the functional brain size between ADHD patients and healthy controls, since studies based on anatomical images reported that the brains of children and adolescents with ADHD were 3–4% smaller than those of children who did not have this disorder (Castellanos et al., 2002). In addition to the total brain size analysis, we also investigated how ADHD affected the functional volume of different brain regions. Finally, we applied the CC400 atlas to calculate the functional volumes for brain regions, and used them as predictors for the classification of ADHD patients vs. TDCs, given that decreased anatomical volume was one of the most replicated pieces of evidence for ADHD (Castellanos et al., 1996, 2001; Berquin et al., 1998; Castellanos, 2002; Mostofsky et al., 2002; Carmona et al., 2005, 2009; Mackie et al., 2007; Wang et al., 2007; Batty et al., 2010; Montes et al., 2011; Qiu et al., 2011; Lopez-Larson et al., 2012). We compared the predictive power of our functional volumes with other relevant feature sets, including regional mean fALFF (Sato et al., 2012b) and anatomical volumes. We also compared our model using functional volumes with the model based on demographic data, since demographic data were shown to outperform resting state fMRI measures in the ADHD-200 global competition (Brown et al., 2012). Additionally, we trained models by integrating functional/anatomical volumes with demographic characteristics, given the possibility that brain volume might be related to personal characteristics and integrating these two types of information might improve the classification performance. Our goal for this study was to verify whether fMRI images could provide additional information that was not included in the anatomical images about the brain volume abnormality in ADHD patients, and consequently lead to a better classification model for the automatic diagnosis of ADHD.

## Materials and methods

### Participants and image acquisition

The ADHD data used in this research was acquired through the public ADHD-200 database through a data use agreement. The ADHD-200 database has de-identified all the patient health information (PHI) associated with the data. No IRB approval for this study is required.

In order to avoid the systematic differences caused by scanner hardware and scanning protocols across different sites (Stonnington et al., 2008; Moorhead et al., 2009; Huppertz et al., 2010; Abdulkadir et al., 2011; Kostro et al., 2014), we used the data from New York University Child Study Center (NYU) only. This center was selected due to its largest sample size among all sites. Although the Peking dataset had a sample size comparable to NYU, the Peking dataset included three batches with different scanning parameters, e.g., different voxel sizes, which may introduce undesirable heterogeneity to the data, and was not used for the present study. The NYU dataset included 263 subjects in total. After subjects whose image quality was questionable for either fMRI images or anatomical images were excluded, 215 subjects were left and were used for the present study. The quality control assessments (usable vs. questionable) based upon visual inspection were provided by ADHD-200 Consortium. One-hundred-and-seventeen of the 215 subjects were ADHD patients (average age = 11.3 years) and the remaining 98 subjects were TDCs (average age = 12.4 years).

For each participant, a high-resolution T1-weighted anatomical image (TI/TR/TE = 1100/2530/3.25 ms; flip angle = 7°; acquisition voxel size = 1.3 mm × 1.0 mm × 1.3 mm; scan time = 8:07 min), and a resting state scan consisting of 176 contiguous whole-brain functional volumes (TR/TE = 2,000/15 ms; flip angle = 90°; acquisition voxel size = 3 × 3 × 4 mm; scan time = 6:00 min) were acquired using a Siemens Magnetom Allegra syngo MR 2004A. Full details about the scanning parameters are available at ADHD-200-Webpage (ADHD-200-Webpage, 2011).

### Preprocessing of images

The anatomical images were preprocessed by our lab using SPM8 with standard procedures. Images were segmented to generate gray matter (GM), white matter (WM), and cerebrospinal fluid (CSF) density maps, which were subsequently normalized to the MNI template with modulation.

The preprocessing of the raw fMRI data was carried out by Neuro Bureau using the Athena pipeline. The Athena pipeline consisted of the following steps:

1. Exclude the first 4 echo-planar (EPI) volumes.
2. Slice timing correction.
3. Deoblique dataset.
4. Motion correction by registering the EPI volumes to the first volume.
5. Spatial normalization.
6. Extract the white matter (WM) and cerebrospinal-fluid (CSF) time-courses.
7. Regress out WM, CSF, motion time courses from EPI data.
8. Temporal band-pass filter (0.009 < f < 0.08 Hz).
9. Spatially smooth the filtered data using a Gaussian filter (full width at half maximum = 6 mm).

After preprocessing, the Neuro Bureau also generated the fALFF maps and made them publicly available at the competition website. For the present study, we used the fMRI brain-only masks (described in Section Introduction), fALFF maps and tissue density maps as the inputs for the machine learning analysis. All the images were spatially normalized to match the MNI template brain.

### Group-level mask

CC400 atlas was generated by the Neuro Bureau using the ADHD-200 dataset. It clustered the voxels into functionally coherent and spatially continuous regions based on the fMRI time courses of the voxels. It could be used as a consensus brain mask for a group of subjects as well as a parcellation of the brain.

### Features

As in Sato et al. (2012b), we employed the CC400 atlas to segment the brain into regions. Since all of the images were normalized to the standard MNI space, voxels from CC400 atlas and voxels from fMRI brain-only masks, fALFF maps or tissue density maps were assumed to be aligned. The parcellation labels in CC400 atlas can be directly transferred to the fMRI brain-only masks, fALFF maps or the tissue density maps. Each region defined in the atlas corresponded to a feature, e.g., a fALFF map can be transformed into a 351-dimensional feature vector using the CC400 atlas. The approaches for calculating the feature values are described below.

#### Functional volume (FV)

For each brain region defined in the CC400 atlas, we calculated the percentage of voxels marked as “1” in the fMRI brain-only mask to quantify the functional volume for this region. Please note the CC400 atlas was a group-level template that was applied to all subjects, while the fMRI masks were generated for each subject individually and they were different for different subjects.

#### Regional mean fALFF

We applied two different approaches to calculate the regional mean fALFF. First, we calculated regional mean fALFF using the traditional method by including all of the voxels covered by the CC400 atlas for each region (Sato et al., 2012b), and denoted it as fALFF1. We noticed that fALFF1 was highly correlated with functional regional volume. For example, a brain region with small functional volume would have many voxels marked as “0” in the fMRI brain-only mask. Those voxels had zero fALFF coefficients and resulted in low regional mean fALFF. Therefore, fALFF1 contained a great deal of information from functional regional volume, which made it difficult to determine whether it was the fALFF information or the functional volume information that was actually relevant to the classification of ADHD patients vs. TDCs. To exclude functional volume information from fALFF1, we calculated fALFF2 by excluding the voxels outside the brain when calculating the regional mean fALFF. Specifically, we calculated the regional mean fALFF over the voxels marked as “1” in the fMRI brain-only mask, given that the fALFF map, fMRI brain-only mask and CC400 atlas were all aligned with each other. This regional mean fALFF was denoted as fALFF2.

#### Anatomical volume

As described in the Section Preprocessing of Images, we obtained three spatially normalized tissue density maps, namely GM, WM, and CSF, for each subject from the segmentation and normalization of the anatomical image. We applied the CC400 atlas to the tissue density maps, and calculated the regional mean tissue density as GM, WM, and CSF volume, respectively.

#### Demographic variables

The NYU dataset provided information for seven demographic variables: gender, age, handedness, verbal IQ, performance IQ, Full4 IQ and medication status. The IQ scores of the participants were evaluated using the Wechsler Abbreviated Scale of Intelligence (WASI). Only the former six demographic variables were used for the present study due to the substantial missing data for medication status. Among the six variables used, only gender was categorical and the remaining five variables were continuous. Gender and age information was available for all 215 participants. There were a couple of missing values for the other four demographic variables. Before imputing the missing values, the six demographic variables were scaled using Equation (1).

(1) $$x^{\prime }=\frac{x-\min(x)}{\max\left(x\right)-\min(x)}$$

The above scaling enabled the different demographic variables to contribute equally during the imputation of missing values. We used the nearest-neighbor method for imputation, with *k* = 10. We also tried *k* = 20, and the imputed data changed only slightly when compared to that generated with *k* = 10. Given a subject with missing data, we calculated the Euclidean distance between this subject and all other subjects based on the demographic variables for which this subject had non-missing values. Then, we found the 10 subjects with the smallest Euclidean distance and calculated a weighted mean for each demographic variable whose value was missing for the current subject based on the values of those 10 neighbors. The weights were calculated as the reciprocals of the Euclidean distances. The weighted mean was subsequently used as the imputed value for the corresponding demographic variable (Hastie et al., 1999; Troyanskaya et al., 2001; Speed, 2003).

### Feature selection

Inspired by previous publications (Saeys et al., 2007; De Martino et al., 2008; Kuncheva and Rodriguez, 2010; Ugurbil et al., 2015) as well as the characteristics of our dataset, we employed a two-step feature selection algorithm by integrating prior knowledge with the recursive feature elimination (RFE) algorithm in this work. As previously mentioned, the functional volume varied across different subjects even though the images had been normalized to the standard template. We speculated that the above variance came from two possible sources: individual differences and ADHD disease status. We would like to exclude the brain regions with only small fluctuations that were likely to be caused by individual difference, because such random fluctuations will not provide any predictive information for the classification of ADHD patients vs. TDCs, and may even confound the classification. In order to select the brain regions that were likely to be affected by ADHD, we calculated the sample variance for each feature/region across different subjects. The features were subsequently sorted according to their variance in descending order. For feature selection, the first step was to select the top N (denoted as *topN*) features with the highest variance, which was essentially the variance threshold feature selection approach (Cui, 2009; Pedregosa et al., 2011). The rationale for doing so was that high variance was likely caused by ADHD rather than random fluctuations. Using the selected features from step one, step two was to perform RFE to pick out the features that were correlated with the class labels. RFE is a popular approach for feature selection. It is commonly used with SVM to repeatedly construct a model and remove features with low weights. There were two parameters in the RFE algorithm: the percentage (*p*) of features to be removed during each iteration, and the number of features to be kept in the final model (*threN*). The algorithm started by training an SVM model using all features selected from step one. Based on the trained model, features were sorted in descending order according to the absolute value of their weights in the SVM model. Features with low absolute weights were removed and a new model with the remaining features was trained. This process was repeated until the number of features reached the predefined threshold *threN*.

The above three parameters were set as follows. Parameter *p* in the RFE algorithm primarily affects the training speed. The smaller the *p*-value, the slower the training and the less likely to remove relevant features. In this project, we set *p* to be 1% and requested the algorithm to remove one feature at a time when the number of features in the model was below 100 (Tan et al., 2015). In order to optimize the parameter *topN* and *threN*, we considered three values for *topN*, namely 100, 200, and 351, and 10 different values for *threN*, from 10 to 100 in steps of 10. Here, we intended to fine-tune the parameter *threN* but not *topN*. The variance threshold was a simple baseline approach based on feature variance and discarded the class label information. It was assumed to be a relatively weak feature selection approach, and served as a preliminary step to reduce the search space for the RFE algorithm. Therefore, the parameter *topN* was not adjusted precisely in order to let the RFE algorithm dominate the feature selection process. The classification performance using different combinations of *topN* and *threN* was summarized in the Results Section.

The above feature selection approaches were applied only to image features. To integrate demographic variables with the image features in the classification model, we treated them differently from the image features: they were always retained in the model, while the image features were ranked and removed based on their variances or weights.

### Classification model learning

The input for model learning was a training set $D=\left\{\left(X_{1},\;y_{1}\right),\cdots ,\left(X_{k},\;y_{k}\right),\cdots ,\left(X_{N},\;y_{N}\right)\right\}$. ***X***_*k*_ = [*x*_1_, ⋯, *x*_*M*_] was the feature vector for the *k*-th sample, *M* was the number of features. *y*_*k*_ = 1 if the *k*-th sample was an ADHD patient, and *y*_*k*_ = 0 if it was a TDC. The classifier we used was the SVM classifier with linear kernel. The basic idea of the SVM classifier was to learn a hyperplane ***wX*** − *b* = 0 to separate ADHD patients from TDCs by maximizing the objective function in Equation (2).

(2) $$\underset{\text{w},\xi ,b}{argmin}\begin{array}{c} \left\{\frac{1}{2}{\left\|\begin{array}{c} \;w \end{array}\right\|}^{2}+C\sum_{k=1}^{N}\xi_{k}\right\}\\ subject\;to\;y_{k}\left(wX_{k}-b\right)\geq 1-\xi_{k},\;\xi_{k}\geq 0\; \end{array}$$

where *C* was a user-defined parameter controlling the trade-off between margin and training errors, and ξ was the slack variable. *C* was set to be 1 in our project according to our previous experiences (Tan et al., 2013, 2015).

After model training, the estimated model is denoted as ŷ = ***wX*** − *b*, which can be used to classify a new sample as either an ADHD patient or a TDC. Given the data (images and demographic information) of a new sample, we first calculate the feature vector ***X*** in the same way as that applied to training samples described in the Section Features, then format **X** to ***X***^***s***^ according to the feature selection results, and finally insert ***X***^***s***^ into the model ŷ = ***wX*** − *b* to obtain a predicted score ŷ for the new sample. If ŷ ≥ 0, the new sample is classified as an ADHD patient. Otherwise, it is a TDC.

### Model evaluation

We employed the 10-fold cross-validation approach for the model evaluation. In this approach, the original samples were randomly partitioned into 10 equal-size subsets. Each time, one subset was used for testing and the remaining nine subsets were used for training. We repeated this process for 10 times, and each subset was used for testing once. The performance statistics were calculated based on the testing samples accumulated from the 10 times of testing. This completed one round of cross-validation. For a fair comparison among different classifiers, e.g., classifiers with different feature sets, we ran 20 rounds of cross-validation for each classifier. The mean performance as well as standard deviation across the 20 rounds of cross-validation were calculated for each classifier, and compared among different classifiers.

Based on the predictions for the testing samples, we calculated sensitivity, specificity, accuracy, and area under receiver operating characteristic curve (AUC) to evaluate the performance of the classifiers. Since the ADHD-200 dataset was an imbalanced dataset, balanced accuracy, which was expressed as (*sensitivity* + *specificity*)/2, was used to accommodate this imbalance in previous studies (Sato et al., 2012a; Lim et al., 2013). Thus, we also calculated the balanced accuracy, and focused on this measure when we compared among different classifiers.

### Identification of important features

Feature importance was measured as the accumulated weights across different folds of cross-validation. Since we had 20 rounds of 10-fold cross-validation, there were in total 200 runs of feature selection and model learning. The importance of a feature was calculated with Equation (3).

(3) $$importance_{i}=\sum_{k=1}^{200}\delta_{i}^{\left(k\right)}|w_{i}^{\left(k\right)}|$$

where $\left|w_{i}^{\left(k\right)}\right|$ was the absolute weight from the SVM model for the *i*th feature during the *k*th run, $\delta_{i}^{\left(k\right)}=1$ indicated that the *i*th feature was selected by the feature selection algorithm during the *k*th run, while $\delta_{i}^{\left(k\right)}=0$ indicated that this feature was not selected.

### Linear regression analysis

A linear regression model has the general form as shown in Equation (4).

(4) Y=Xβ+ ε

Let *n* denote the number of subjects and *p* denote the number of covariates. Then *Y* is an *n*-dimensional vector of a continuous response variable, *X* is the design matrix whose dimension is *n* × *p*, β is the *p*-dimensional vector of coefficients to be estimated, and ε is an *n*-dimensional vector of random errors. Generally, we have Gauss–Markov assumptions on the random error vector ε. It states that all random errors ε_*i*_ (*i* = 1, …, *n*) are mutually independent and have constant variance σ^2^ and zero mean. Under this assumption, the best linear unbiased estimator of β is $\hat{\beta }=\;{\left(X^{\prime }X\right)}^{-1}X^{\prime }Y$, which can be obtained using ordinary least square. The variance of $\hat{\beta }\;$is σ^2^(*X*′*X*)^−1^. In order to make inference or perform statistical testing, we often impose normality assumption on the random errors, i.e., ε ~ *N*(0, σ^2^*I*), where *I* is the identity matrix. Under normality assumption, we can perform statistical tests on any coefficient in the model. Generally we use *t*-test to test whether a single coefficient equals to zero or not. It has the form $T=\;\frac{{\hat{\beta }}_{i}}{SE\left({\hat{\beta }}_{i}\right)}=\;\frac{{\hat{\beta }}_{i}}{\sqrt{{\hat{\sigma }}^{2}c_{ii}}}$, where *T* is the t-statistic, *c*_*ii*_ is the *i*-th diagonal element of matrix (*X*′*X*)^−1^, and ${\hat{\sigma }}^{2}=\frac{1}{n-p}\overset{n}{\underset{k=1}{\sum }}{\left(Y-Ŷ\right)}^{2}$.

Using the above linear regression model, we compared the functional brain size between ADHD patients and TDCs. Similar to the calculation of functional regional volume, functional brain size for a subject was calculated as the percentage of “1” voxels within the whole brain. Using the brain size calculated from the fMRI brain-only masks, we analyzed the effect of ADHD on the brain size. We performed a linear regression analysis as described above with brain size as the response variable and the ADHD index as one covariate. ADHD index was an overall measure of symptom severity. The higher the ADHD index, the more severe the symptom is. We used ADHD index instead of ADHD diagnosis labels because binarization results in some information loss. Additionally, personal characteristics, such as age and gender, may also affect the brain size. Therefore, we included the six demographic variables in the linear model as well.

In order to analyze how ADHD affected different brain regions, we performed a linear regression analysis for each single brain region. The analysis was the same as the brain size analysis except that we used the functional regional volume instead of the functional whole brain size as the response variable. The predictors still included the ADHD index and the six demographic variables as above.

## Results

### Linear regression analysis results

For the functional brain size analysis, the linear regression model was summarized in Table 1. As expected, the coefficient for ADHD index was negative, which indicated that the more severe the ADHD symptoms the subjects had, the smaller their brains were. However, the coefficient for the ADHD index was not significantly different from zero according to the *t*-test results. Handedness and IQ scores were also showed to be non-significant. On the other hand, age and gender exhibited significant influence on the functional brain size, although the images were normalized to the standard template. Due to their significant effects on brain size, it was reasonable to integrate the demographic variables or at least age and gender with the functional volume features. A model integrating these two types of information was likely to have an improved classification performance.

**Table 1:** Functional brain size analysis: a summary for the linear regression model.

**Variable**	**Coefficient Estimate**	**Std. Error**	***p*-value**
Intercept	0.622	0.152	6.12e-5
ADHD index	−0.045	0.041	0.273
Gender	0.060	0.021	0.005^*^
Age	−0.122	0.039	0.002^*^
Handedness	0.104	0.061	0.092
Verbal IQ	−0.350	0.559	0.531
Performance IQ	−0.278	0.505	0.583
Full4 IQ	0.576	0.881	0.514

* *We use 0.05 as significance level*.

Among the 351 brain regions defined in the CC400 atlas, there were 77 regions whose volumes were exactly the same across all of the subjects. We assumed that there was no disease effect on those regions, and excluded them from our analysis. Therefore, we performed a linear regression analysis for each of the 274 remaining regions and employed the False Discover Rate (FDR) procedure (Benjamini and Hochberg, 1995) to adjust the *p*-value for multiple testing. Twenty-six of the 274 analyzed brain regions showed significant disease effect, i.e., uncorrected *p*-value for the coefficient of ADHD index was below 0.05. None of the 26 regions survived through *p*-value correction using the FDR procedure. In our project, however, traditional *p*-value correction methods might be too stringent for two reasons. First, some brain regions had only subtle variance in functional volume across different individuals, which was likely to be random noise caused by individual difference instead of actual change in functional volume caused by disease. We were not likely to detect significance for those brain regions. Including those brain regions in the analysis, however, increased the number of multiple testing, which would decrease the discovery rate for other regions. Secondly, there were correlations between different brain regions. Therefore, FDR procedure (Benjamini–Hochberg) which relied heavily on independence assumption might give a too stringent control (Benjamini and Hochberg, 1995). There were some other multiple testing procedures for dependence hypothesis (Benjamini and Yekutieli, 2001; Sun and Cai, 2009), but it was difficult to verify whether they would truly work for our problem since the dependence structure of different brain regions was unknown. We therefore decided not to implement those methods.

Besides, we noticed that 23 out of the above 26 brain regions (uncorrected *p* < 0.05) had negative coefficients in the linear regression analysis. Six out of those 26 regions had uncorrected *p*-value below 0.01. All of these six regions had negative coefficients too. They were adjacent to each other and located in the frontal lobe exclusively. Those observations suggested that ADHD might indeed cause brain shrinkage, but its effect on each single brain region was subtle, and frontal lobe might be one of those regions that showed relatively high severity.

### Classification performance without feature selection

The classification performance without feature selection was summarized in Table 2. The model using demographic characteristics alone achieved a balanced accuracy of 58.5%, which surpassed all image feature sets except for functional volume (FV) that had a balanced accuracy of 59.6%. We compared the performance of the model with demographic characteristics with the performance of the model with FV using Student's *t*-test. No significance was detected (two-sided *p* = 0.10). Comparisons between different models in the rest of this manuscript were also done using Student's *T*-test, and the reported *p*-values were two-sided *p*-values. Integrating the demographic variables with image features helped to improve the classification performance when compared to image features alone, expect for fALFF1. Nevertheless, none of the integrated feature sets, such as fALFF1+Demo and fALFF2+Demo (Table 2), outperformed the demographic characteristics alone, except for FV+Demo which showed significantly better performance than demographic characteristics alone with *t*-test *p* = 1.2e-4. FV outperformed both fALFF1 and fALFF2, especially after integrating demographic information into the model. Compared with fALFF2, fALFF1 achieved better performance, which was expected since fALFF1 included a great deal of FV information as explained in Section Regional Mean fALFF. fALFF2 without integrating demographic information exhibited almost random classification with AUC and balanced accuracy around 0.5, which was much lower than that of fALFF1 and FV. This might indicate that FV encoded most of the predictive information, while fALFF itself had very limited predictive power. Furthermore, FV also demonstrated better performance than anatomical features (GM/WM/CSF). In summary, functional volume exhibited the highest discriminative power, outperforming the demographic variables, fALFF features and anatomical features.

**Table 2:** Classification performance without feature selection.

**Feature Set**	**sens. (%)**	**spec. (%)**	**accu. (%)**	**AUC**	**(sens+spec)/2 (%)**
Demo	**70.9** ± **2.4**	46.2 ± 1.8	59.6 ± 1.6	**0.65** ± **0.02**	58.5 ± 1.6
FV	67.7 ± 3.0	51.6 ± 2.7	60.3 ± 2.1	0.62 ± 0.02	59.6 ± 2.1
fALFF1	63.5 ± 3.1	51.9 ± 2.4	58.2 ± 2.3	0.60 ± 0.02	57.7 ± 2.3
fALFF2	64.2 ± 2.5	39.7 ± 3.2	53.0 ± 1.7	0.52 ± 0.02	52.0 ± 1.8
GM	63.3 ± 1.8	46.7 ± 3.8	55.7 ± 1.8	0.56 ± 0.02	55.0 ± 2.0
WM	56.9 ± 2.5	47.7 ± 2.6	52.7 ± 2.1	0.51 ± 0.02	52.3 ± 2.1
CSF	56.4 ± 2.8	39.6 ± 3.4	48.7 ± 2.7	0.49 ± 0.03	48.0 ± 2.8
FV+Demo	68.2 ± 3.3	**54.5** ± **2.4**	**62.0** ± **2.3**	0.64 ± 0.02	**61.4** ± **2.2**
fALFF1+Demo	62.5 ± 2.5	52.4 ± 2.7	57.9 ± 2.0	0.62 ± 0.02	57.4 ± 2.0
fALFF2+Demo	62.3 ± 2.6	44.1 ± 3.6	54.0 ± 2.4	0.58 ± 0.02	53.2 ± 2.5
GM+Demo	62.8 ± 2.4	51.7 ± 2.1	57.7 ± 1.6	0.61 ± 0.01	57.3 ± 1.6
WM+Demo	58.3 ± 2.2	50.2 ± 2.5	54.6 ± 1.9	0.55 ± 0.02	54.2 ± 1.9
CSF+Demo	62.1 ± 3.0	49.2 ± 2.8	56.2 ± 2.2	0.56 ± 0.02	55.6 ± 2.2

*Demo is short for demographic variables; FV is short for functional volume; fALFF1/fALFF2 is short for regional mean fALFF1/fALFF2; GM/WM/CSF is short for GM/WM/CSF volume; X+Demo is short for the combination of X feature set and demographic variables. The best sens., spec., accu., AUC and (sens+spec)/2 are marked by bold*.

### Classification performance with feature selection

**Figure 2:**
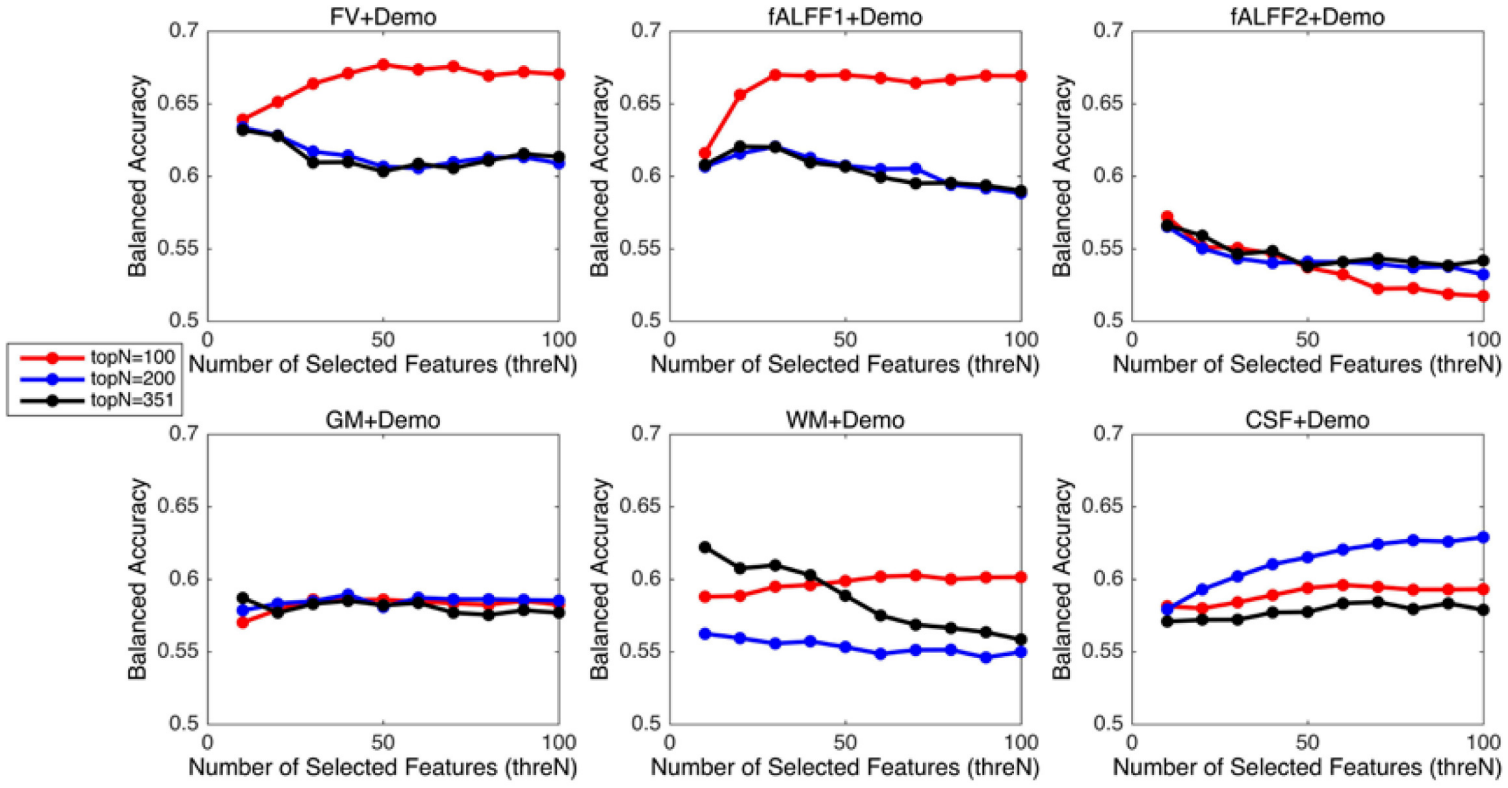
Classification performance with feature selection using different parameters.

Since integrating demographic variables tended to improve the classification performance, we only considered the models integrating demographic variables for this section. Performance under different parameters was shown in Figure 2. FV and fALFF1 exhibited similar pattern. The performance of *topN* = 200 and *topN* = 351 were close to each other, but obviously worse than that of *topN* = 100, no matter what the value of *threN* was. *topN* = 351 represented the situation where we did not preselect the brain regions with high variance and submitted all the features to the RFE process, which was actually the standard RFE. Our results suggested that a pre-selection based on variance before RFE indeed helped to improve the classification performance. As we explained previously, we did not test all range of *topN* but considered only three different values for *topN*. It was likely that there were fewer noise features disturbing the RFE algorithm when *topN* = 100. Thus, it was understandable that the performance with *topN* = 100 was much better than the performance with *topN* = 200 or 351. When *topN* = 100, the performance tended to reach a relatively stable status when *threN* = 50 for FV and *threN* = 30 for fALFF1. We reported the classification performance in Table 3 for FV with *topN* = 100, *threN* = 50 as well as the performance for fALFF1 with *topN* = 100, *threN* = 30. For all of the other feature sets including fALFF2, GM, WM, and CSF, we reported their best performance across all the combinations of *topN* and *threN* in Table 3. Clearly, this might overestimate the actual classification performance for those four feature sets. Nevertheless, the performance for those four features sets was still much worse than the performance of FV and fALFF1. We also compared each model with the model of FV+Demo using *t*-test. The two-sided *p*-values were shown in Table 3.

**Table 3:** Classification performance with feature selection.

**Feature Set**	**sens. (%)**	**spec. (%)**	**accu. (%)**	**AUC**	**(sens+spec)/2 (%)**	***p*-value**
FV+Demo	**78.1** ± **2.9**	57.3 ± 2.3	**68.6** ± **1.7**	0.71 ± 0.01	**67.7** ± **1.7**	NA
fALFF1+Demo	75.2 ± 2.2	**58.8** ± **2.5**	67.7 ± 1.5	**0.72** ± **0.02**	67.0 ± 1.5	0.12
fALFF2+Demo	66.1 ± 3.3	48.4 ± 3.4	58.0 ± 2.8	0.64 ± 0.02	57.2 ± 2.8	3.22E-12
GM+Demo	67.0 ± 3.2	50.9 ± 2.4	59.7 ± 2.3	0.64 ± 0.02	58.9 ± 2.2	1.13E-12
WM+Demo	69.5 ± 3.1	54.9 ± 4.4	62.9 ± 3.0	0.66 ± 0.02	62.2 ± 3.1	2.05E-7
CSF+Demo	70.1 ± 1.8	55.7 ± 1.9	63.5 ± 1.4	0.67 ± 0.02	62.9 ± 1.4	1.25E-8

*The best sens., spec., accu., AUC and (sens+spec)/2 are marked by bold*.

### Important features

**Figure 3:**
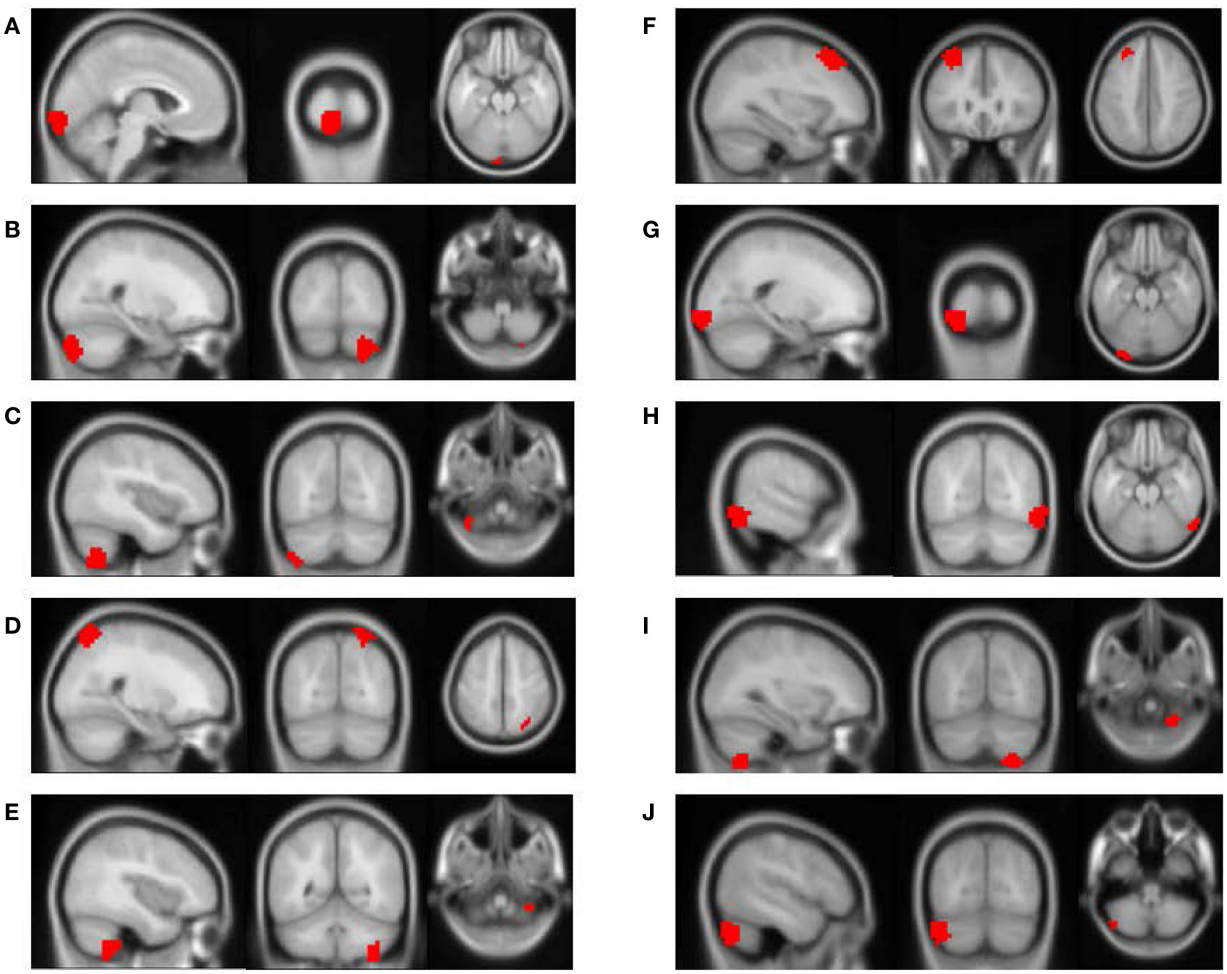
The top 10 discriminative brain regions distinguishing between ADHD patients and TDCs. Images were displayed in neurological orientation using xjView toolbox (http://www.alivelearn.net/xjview). **(A)** Region 1, **(B)** Region 2, **(C)** Region 3, **(D)** Region 4, **(E)** Region 5, **(F)** Region 6, **(G)** Region 7, **(H)** Region 8, **(I)** Region 9, **(J)** Region 10.

**Figure 4:**
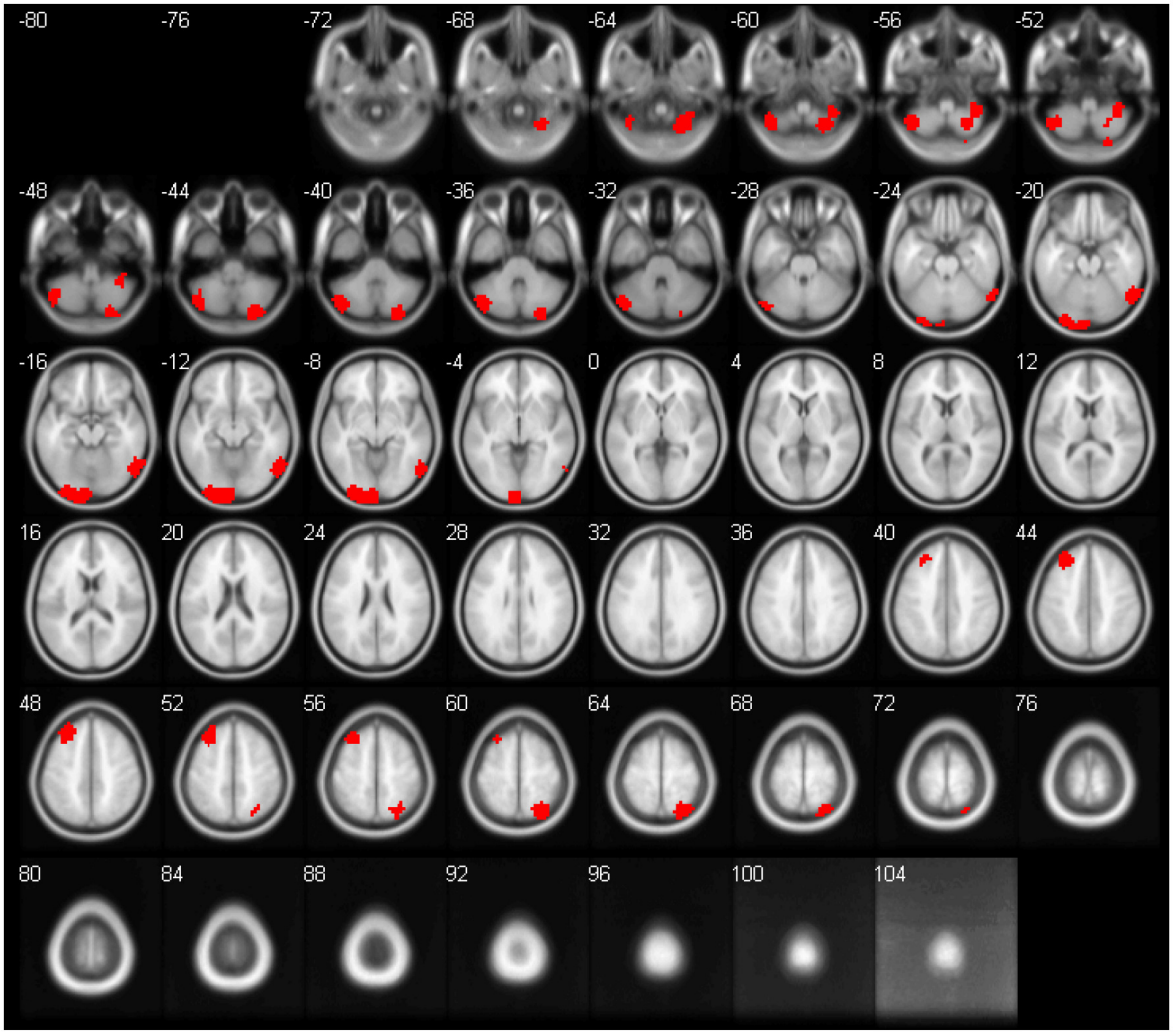
The top 10 regions in Figure 3 were combined and displayed in the brain space simultaneously.

The feature importance was calculated based on the model integrating FV and demographic variables. Feature selection was performed with *topN* = 100 and *threN* = 50. The feature importance was calculated using Equation (3). Features were ranked according to their importance in descending order. We projected the top 10 features to the brain space as shown in Figure 3. The anatomical information for those 10 brain regions was summarized in Table 4. Since some important regions, e.g., regions A and G, appeared to be adjacent to each other, we marked the top 10 regions in a single brain, as shown in Figure 4. The predictive regions were mainly located in occipital lobe, cerebellum posterior lobe, parietal lobe, frontal lobe, and temporal lobe.

**Table 4:** Anatomical information for the top 10 discriminative brain regions displayed in Figure 3.

**Region index**	**Central coordinates**	**Anatomical location**
A	(−4, −100, −22)	Left occipital lobe
B	(24, −84, −54)	Right cerebellum posterior lobe
C	(−40, −68, −62)	Left cerebellum posterior lobe
D	(24, −72, 54)	Right parietal lobe
E	(40, −52, −62)	Right cerebellum posterior lobe
F	(−32, 28, 42)	Left frontal lobe
G	(−20, −100, −22)	Left occipital lobe
H	(60, −68, −22)	Right temporal lobe
I	(32, −68, −66)	Right cerebellum posterior lobe
J	(−48, −76, −46)	Left cerebellum posterior lobe

Besides, we checked the correspondence between the top 10 regions in Figure 3 and the top 10 brain regions in the linear regression analysis. Only the brain region with the highest discriminative power (Region A in Figure 3) was also ranked among top 10 in the linear regression analysis. This result was not unusual. The linear regression analysis considered one brain region at a time and ignored the correlations between different brain regions, e.g., the top 6 regions (uncorrected *p* < 0.01) were generally from the same brain area. Furthermore, our linear regression analysis suggested that the effect of ADHD on each single brain region was subtle. Few brain regions were strong enough to be necessarily selected by the RFE algorithm and weighted heavily by the SVM model. On the other hand, the RFE algorithm and the SVM model considered multiple brain regions simultaneously. It was very likely that the combination of two or more brain regions provided significant discriminative power, but each of them individually only exhibited limited distinction between ADHD patients and healthy controls. Therefore, it was not surprising to see that the top regions from the SVM model were not highly ranked in the linear regression analysis.

## Discussion

In the present study, we have initially proposed the concept of functional volume. We compared the discriminative power of functional volume with that of anatomical volume in the task of classifying ADHD patients from TDCs. Results showed that the functional volume obtained better performance, but the underlying reason still needs further investigation. One possible explanation is that necrotic or functionally deficient brain tissues might exist in some brain areas. Such necrotic tissues can be captured by anatomical images, and prevent an accurate measure of brain volume using anatomical images. By contrast, functional volume would automatically exclude such necrotic tissues, because they do not have fMRI signal. Thus, compared to anatomical volume, functional volume may serve as a better way of measuring active brain volume. Although the current hypothesis requires future experimental validation, our present study provided a new perspective for measuring brain volume and its shrinkage, which might encourage future experimental studies in related fields.

Another major contribution of this paper was that we had improved the accuracy of ADHD diagnosis. Although functional volume had comparable performance as fALFF1, our study shed light on why the regional mean fALFF distinguished between ADHD patients and TDCs. According to our analysis, it was the functional volume other than fALFF itself that encoded the discriminative information. Upon this observation, we made two major improvements based on the model in Sato et al. (2012b). The first improvement was to integrate the demographic information with the regional mean fALFF. Since the regional mean fALFF mainly captured brain volume information and brain volume was likely to be related to demographic characteristics, integration of these two types of information became straightforward. Otherwise, combination of fALFF maps with demographic characteristics might be less intuitive. Inspired by the observation in this paper, we designed a two-step feature selection algorithm by integrating the variance threshold approach with the standard RFE algorithm. This two-step algorithm worked well for both functional volume and fALFF1, and performed much better than the standard RFE algorithm. The above two improvements helped to improve the balanced accuracy from 57.7 to 67.0% for the fALFF1 feature set. Finally, Sato et al. (2012b) reported that combination of ReHo and fALFF contained relevant information to discriminate ADHD patients from TDCs. They compared 10 different classifiers and suggested that all classifiers provided nearly the same performance for the classification of ADHD patients vs. TDCs. For comparison purposes, we also calculated the regional mean ReHo and fALFF using the CC400 atlas as in Sato et al. (2012b), and then used them as features to train a linear SVM model. The model was evaluated in the same way as all of the other models in this paper. This SVM model based on ReHo and fALFF achieved a balanced accuracy of 51.6%, which was 16.1% lower than our model using functional volume with feature selection. Also, please note that the combination of ReHo and fALFF performed worse than fALFF alone whose balanced accuracy was 57.7% for our dataset (Table 2). Although Sato et al. suggested that the combination of ReHo and fALFF was the best feature set for the classification of ADHD patients vs. TDCs, the best balanced accuracy they achieved was 53.6%, which was relatively low and did not show significant superiority over fALFF alone. While they used the whole ADHD-200 dataset accumulated from the eight study centers, we used only the dataset from the NYU study center. With a different dataset in our current study, it seemed that the ReHo features did not bring in additional power to our classification problem, but added noise that disrupted our model, given that feature selection was not applied.

We also applied our model on the dataset from another ADHD-200 study center, namely Oregon Health & Science University (OHSU). The classification performance for different models was shown in Table S1. The model with demographic variables alone classified all of the subjects as healthy controls. Sato's model (Sato et al., 2012b), combining ReHo and fALFF, achieved a balanced accuracy 58.7%. Regional fALFF without demographic data and feature selection achieved a balanced accuracy 60.9%. Consistent with the results on NYU dataset as analyzed above, integration with demographic data and our feature selection algorithm helped to improve the balanced accuracy to 69.5% for regional fALFF. Unlike NYU results, however, fALFF2 achieved relatively good results for the OHSU dataset, which indicated that both functional volume and regional fALFF included some discriminative information to distinguish ADHD patients from healthy controls for the OHSU dataset. This was also the underlying reason why fALFF1+Demo outperformed FV+Demo for this dataset.

Although, CC400 atlas was clearly a functional atlas generated from fMRI data, it was also used for the anatomical images in this project. For a fair comparison, we had tried the Automated Anatomical Labeling (AAL) atlas (Tzourio-Mazoyer et al., 2002) for the GM, WM and CSF maps. The AAL atlas was defined based on brain anatomy. It divides the brain into 116 regions, including 90 cerebrum regions and 26 cerebellum regions. We calculated the regional mean tissue density as the features. The approach to calculate the feature values was the same as the approach described in paragraph ***Anatomical Regional Volume***, except replacing the CC400 atlas with the AAL atlas. The model training and evaluation was the same as the other models in this paper. The classification performance without feature selection was shown in Table S2. For feature selection, we applied the standard RFE algorithm, since there were only 116 features. The percentage of features to be removed at each iteration was set at 1% and the number of features to be kept in the final model was set to be 10–100, stepping by 10. The best classification performance across different parameters was shown in Table S3. As we can see, performance for the anatomical features using AAL atlas was still worse than the performance of functional volume features. Further, CC400 atlas might not be the optimal way to segment the brain for calculating functional volumes either. As shown in Figure 4, the top 10 discriminative regions were adjacent to each other, suggesting that the regions could be further merged into larger regions. A brain segmentation characterizing the functional shrinkage pattern in brains of ADHD patients may further improve the classification performance for the functional volume features. Investigating the optimal way of segmenting the brain to maximize the classification performance might be a future direction of work.

Our classification algorithm detected functional volume differences in occipital lobe, cerebellum posterior lobe, parietal lobe, frontal lobe, and temporal lobe, including several brain regions that have been consistently implicated across studies or have been shown to have a large between-group effect size in anatomical analyses. For example, Valera et al. (2007) found that the brain region with the largest reduction in patients with ADHD compared to controls was the cerebellum, both in specific regions (i.e., inferior vermis) as well as more globally (i.e., both right and left cerebellum). Another common finding in ADHD structural imaging studies is that patients with ADHD have globally reduced gray matter in the cortices (Shaw et al., 2006; Valera et al., 2007). However, these reductions in cortical gray matter are often found to be greatest in frontal regions (Valera et al., 2007). In addition, sub-cortical structures, such as caudate, putamen, and globus pallidus, have repeatedly been found to be reduced in patients with ADHD (Ellison-Wright et al., 2008; Nakao et al., 2011; Frodl and Skokauskas, 2012), but they did not appear among the top 10 brain regions with highest discriminative power. To check the underlying reason why the sub-cortical structures were not identified, we checked the brain regions ranked from 11 to 20, and noticed that the region ranked 19 involving sub-cortical structures such as caudate and putamen. Furthermore, all except one of the regions ranked from 11 to 18 came from Cerebellum Posterior Lobe/Frontal Lobe/Temporal Lobe that had already been listed in Table 4. Therefore, the sub-cortical structures also appeared to be an important region distinguishing between ADHD patients and health controls based on functional volume. There might be additional regions affected by ADHD, but have not been identified as the top regions by our algorithm. Particularly, interior regions of the brain might be more likely to be under-estimated than regions near the surface of the brain, because interior regions tended to have a functional volume of 100% or nearly 100% after spatial normalization. Future work needs to be done to remove this limitation. Aside from the cerebellum posterior lobe and frontal lobe, the occipital lobe, parietal lobe, and temporal lobe were also identified as top discriminative regions. Although, previous ADHD morphometry research has implicated nearly the entire cerebral cortex as well as many brain substructures (e.g., caudate) as being smaller or less developed in children with ADHD than in controls, there were not many studies focused on those three regions to the best of our knowledge. Our current study might provide some new perspectives for the experimental researchers and encourage future studies for those regions.

Another concern for the current analysis might be that functional volume could be strongly driven by movement artifacts. The Neuro Bureau organization had taken critical steps, e.g., motion correction and regression out motion time courses, to remove the effects of head motions as much as possible during the preprocessing of images. Furthermore, we did a correlation analysis between the head motions and the functional volumes. The Neuro Bureau organization provided the max motion for each subject, which was calculated as the maximal movement in three directions across all time points during the fMRI imaging. We calculated the Spearman's correlation *r*- and *p*-value between the max motion and the functional volumes for the top 4 discriminative regions listed in Table 4. We did not detect any significant correlations as shown in Figure S1.

## Conclusion

This study started out to build an accurate classifier for the automatic diagnosis of ADHD. We proposed to quantify the functional volume of the whole brain or a brain region using the brain-only mask generated by the AFNI program “3dAutomask” based on fMRI data. The functional brain size measured from the fMRI mask exhibited significant correlations with demographic characteristics such as age and gender, although the images had been normalized to the standard template. This result not only pointed to the weakness of current spatial normalization algorithm, but also demonstrated that it is essential to control those personal variables while comparing the functional brain size between different groups of individuals, e.g., comparing the functional brain size of the patient group with that of healthy controls. Otherwise, the analysis result might be misleading. Furthermore, we calculated the functional volumes for the brain regions defined by CC400 atlas, and used them as features for the classification of ADHD patients vs. TDCs. Functional volumes demonstrated much higher discriminative power than anatomical volumes which were calculated in the same way using the tissue density maps. With our two-step feature selection algorithm, the model based on functional volumes also exhibited much better classification performance in comparison with related models in literature. Finally, our results also demonstrated that fALFF itself had very limited predictive power for the classification of ADHD patients vs. TDCs. The regional mean fALFF calculated using the traditional method was highly correlated with functional volume. Caution should be applied when we attempt to compare the fALFF between different groups of individuals, especially when there is brain shrinkage for one group of individuals. In fact, this observation is not limited to fALFF alone. Any voxel-wise features, such as ReHo, would have the same problem and should be given particular attention when comparing between different groups of individuals. It might be not fair to claim that some brain regions have decreased fALFF or ReHo, if the decreases in fALFF/ReHo are in fact caused by the reduced brain volume.

## Author contributions

LT conceived the project idea and performed the experiments. LL supervised the project. XG and SR provided critical suggestions for the statistical analysis. JE surveyed the literature to check whether our findings of the discriminative brain regions were consistent with previous publications. All authors were involved in writing and preparing the manuscript.

### Conflict of interest statement

The authors declare that the research was conducted in the absence of any commercial or financial relationships that could be construed as a potential conflict of interest.
