# Supplementary material for: A Computational Model for the Automatic Diagnosis of Attention Deficit Hyperactivity Disorder Based on Functional Brain Volume
Source: Front Comput Neurosci. 2017 Sep 8;11:75. doi: 10.3389/fncom.2017.00075 (PMC5596085; doi:10.3389/fncom.2017.00075)
Supplement: Supplementary file 1 [file Table1.pdf]

Table S1. Classification performance on dataset from Oregon Health & Science University (OHSU)

features	with feature selection	sens.(%)	spec.(%)	accu.(%)	AUC	(sens+spec)/2(%)
Demo	No	0.0±0.0	100.0±0.0	50.0±0.0	0.56±0.03	50.0±0.0
Sato's method	No	43.7±3.6	73.7±3.2	63.5±2.6	0.63±0.03	58.7±2.6
fALFF1	No	45.3±4.8	76.4±2.6	65.9±2.6	0.65±0.03	60.9±3.0
FV+Demo	Yes	45.9±4.1	86.5±2.6	72.7±2.8	0.68±0.02	66.2±3.1
fALFF1+Demo	Yes	50.2±4.0	88.9±3.1	75.7±2.8	0.72±0.03	69.5±2.9
fALFF2+Demo	Yes	35.3±3.4	92.9±1.6	73.3±1.5	0.71±0.02	64.1±1.8
