# Supplementary material for: A Computational Model for the Automatic Diagnosis of Attention Deficit Hyperactivity Disorder Based on Functional Brain Volume
Source: Front Comput Neurosci. 2017 Sep 8;11:75. doi: 10.3389/fncom.2017.00075 (PMC5596085; doi:10.3389/fncom.2017.00075)
Supplement: Supplementary file 2 [file Table2.pdf]

Table S2. Classification performance of anatomical volume using AAL atlas without feature selection

features	sens. (%)	spec. (%)	accu. (%)	AUC	(sens+spec)/2 (%)
GM	**70.3±2.7**	42.4±2.5	57.6±1.7	0.57±0.01	56.4±1.7
WM	66.4±2.3	44.2±2.9	56.3±2.0	0.56±0.02	55.3±2.1
CSF	67.3±2.2	34.5±2.4	52.3±1.6	0.54±0.02	50.9±1.6
GM+Demo	67.1±2.0	49.6±2.1	59.1±1.5	**0.65±0.01**	58.3±1.5
WM+Demo	65.5±1.7	**52.9±2.7**	59.8±1.9	0.63±0.01	59.2±1.9
CSF+Demo	67.1±2.6	51.9±2.9	**60.2±2.5**	0.64±0.02	**59.5±2.5**
