# Supplementary material for: A Computational Model for the Automatic Diagnosis of Attention Deficit Hyperactivity Disorder Based on Functional Brain Volume
Source: Front Comput Neurosci. 2017 Sep 8;11:75. doi: 10.3389/fncom.2017.00075 (PMC5596085; doi:10.3389/fncom.2017.00075)
Supplement: Supplementary file 3 [file Table3.pdf]

Table S3. Classification performance of anatomical volume using AAL atlas with feature selection

features	sens. (%)	spec. (%)	accu. (%)	AUC	(sens+spec)/2 (%)
GM+Demo	**67.8±1.9**	49.9±2.5	59.7±1.7	**0.65±0.01**	58.9±1.7
WM+Demo	66.0±2.6	**53.0±2.6**	60.0±2.1	0.64±0.01	59.5±2.1
CSF+Demo	67.2±2.1	52.4±2.7	**60.4±2.1**	0.63±0.02	**59.8±2.1**
