# Supplementary figures and images for: A Computational Model for the Automatic Diagnosis of Attention Deficit Hyperactivity Disorder Based on Functional Brain Volume
Source: Front Comput Neurosci. 2017 Sep 8;11:75. doi: 10.3389/fncom.2017.00075 (PMC5596085; doi:10.3389/fncom.2017.00075)

**Figure S1.** Correlation between head movements and functional volumes.

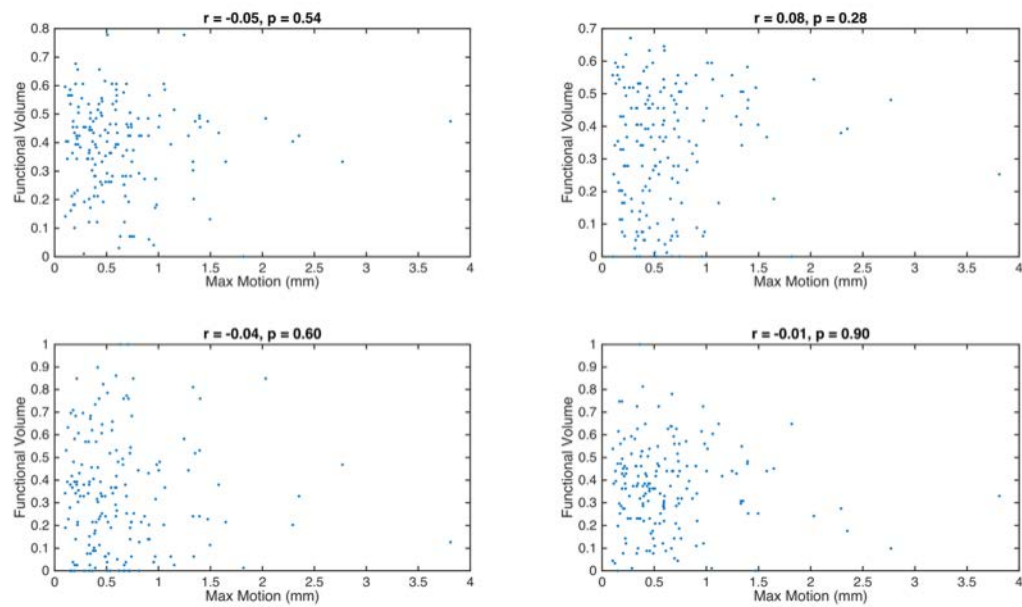

Supplement: Supplementary file 4 [file Image1.pdf]
